# Epidermal Growth Factor Receptor Family and its Role in Gastric Cancer

**DOI:** 10.3389/fonc.2019.01308

**Published:** 2019-11-26

**Authors:** Chiara Arienti, Sara Pignatta, Anna Tesei

**Affiliations:** Biosciences Laboratory, Istituto Scientifico Romagnolo per lo Studio e la Cura dei Tumori (IRST) IRCCS, Meldola, Italy

**Keywords:** HER2, EGFR, tyrosine kinase inhibitor, targeted therapy, gastric cancer, clinical trial

## Abstract

Despite the gradual decrease in incidence, gastric cancer is still the third leading cause of cancer death worldwide. Although chemotherapy enhances overall survival and quality of life in advanced disease, the median overall survival is < 12 months. In recent years, the human epidermal growth factor receptor (ErbB) family has been extensively investigated in gastric cancer. The ErbB family is composed of four closely-related members: ErbB-1 (HER1 or epidermal growth factor receptor, EGFR), ErbB-2 (HER2), ErbB-3 (HER3), and ErbB-4 (HER4), all of which play a critical role in regulating cell growth, proliferation and migration of tumors. It is well known that gastric cancer overexpresses HER in a heterogeneous pattern, especially EGFR, and HER2. HER3 is another important member of the ErbB family that preferentially activates the phosphatidylinositol 3-kinase (PI3K) pathway. Furthermore, its heterodimerization with HER2 seems fundamental for steering HER2-overexpressing breast cancer tumor growth. Less is known about the impact of HER4 on gastric cancer. Improved survival from the use of trastuzumab has paved the way for ErbB receptor family-targeted treatments in gastric cancer. However, unlike trastuzumab, ErbB receptor-targeted drugs have not consistently maintained the encouraging results obtained in preclinical and early clinical trials. This may be attributable to the intrinsic heterogeneity of gastric cancer and/or to the lack of standardized test quality for established biomarkers used to evaluate these biological targets. This review presents an overview of the most recent clinical studies on agents targeting the ErbB family in gastric cancer.

## Introduction

Gastric cancer is the fifth most common malignancy and the third leading cause of cancer death worldwide (1). In Europe, it is the fifth most common cancer in both sexes, accounting for around 23% of all cancers. The annual incidence is 20/100,000 for men and 9/ 100,000 for women, resulting in ~107,000 deaths annually (2). There is a distinct geographic variability in gastric cancer, the highest rates being observed in East Asia, South America and Eastern Europe, and the lowest rates in the U.S. and Western Europe (3).

## Treatment Options for Gastric Cancer

### Surgery

Surgical resection remains the primary treatment for all patients with regionally confined disease, the extent of the intervention depending on the site of the tumor. Total gastrectomy is usually recommended for proximal tumors but is not considered superior to subtotal gastrectomy in terms of survival in distal gastric cancer (4, 5).

Patients with superficial early gastric cancer (T1a) are candidates for endoscopic mucosal resection (EMR). T1a is defined as adenocarcinomas confined to the mucosa, <2 cm in diameter, low-moderate differentiation, no evidence of ulcer, and with no lymphovascular involvement (6, 7). The extent of lymph node dissection is hotly debated and studies have failed to confirm a survival benefit of D1 dissection (dissection of the perigastric nodes) over D2 dissection (dissection of perigastric nodes and nodes along the left gastric, hepatic, celiac, and splenic arteries). D2 dissection is associated with fewer locoregional recurrences and gastric cancer-related death, but also with higher rates of morbidity and mortality. A modified (spleen-preserving) D2 dissection is considered standard treatment in many hospitals. The addition of para-aortic dissection to D2 dissection does not improve survival (8–10).

Surgical resection represents standard curative gastric cancer treatment (11), but around 25% of patients have unresectable tumors at diagnosis due to the presence of metastatic disease.

### Chemotherapy or Chemoradiation

Chemotherapy is considered a feasible option in patients with metastases but good functional status and an acceptable life expectancy (12, 13). First-line treatment for metastatic disease includes a combination of a platinum compound and fluorouracil. The addition of another agent such as an anthracycline (e.g., epirubicin) in Europe (ECF = epirubicin, cisplatin, and fluorouracil) or a taxane (e.g., docetaxel) in the U.S. (DCF = docetaxel, cisplatin, and fluorouracil) is common practice (14–16). Other studies have demonstrated that fluorouracil can be substituted by capecitabine, and cisplatin by oxaliplatin (17, 18). Substituting oxaliplatin for cisplatin is associated with lower toxicity (17, 19). Recent trials have used EOX (epirubicin, oxaliplatin, and fluorouracil) and FLO (fluorouracil, calcium folinate [leucovorin], and oxaliplatin) (17, 18).

Overall survival is higher in patients with locally advanced gastric cancer treated with chemoradiation than in those treated with radiation alone (20, 21). Adverse effects of radiation include nausea, vomiting (patients may need to be pre-treated with antiemetics prior to radiation), weight loss, and diarrhea. Less commonly, radiation can cause small bowel obstruction, liver damage, and kidney damage.

### Perioperative Chemoradiation or Chemotherapy

In patients with pathologic stage II-IIIC or any T, N+ disease or R1 resection, postoperative radiation combined with adjuvant fluorouracil has been shown to improve overall survival (22, 23). Postoperative chemoradiation consists of one cycle of fluorouracil (with or without calcium folinate) given prior to radiation, followed by two cycles after radiation. During radiation, patients receive fluorouracil on the first 4 and last 3 days of radiation. Preoperative chemoradiation consisting of radiotherapy and fluorouracil (or fluorouracil and paclitaxel) is used to induce tumor downstaging and increase respectability (24). Another option is adjuvant chemotherapy, which improves survival in patients undergoing curative resection (25, 26). In patients with stage II or higher gastric cancer, perioperative chemotherapy with ECF (epirubicin, cisplatin, and fluorouracil) has been shown to improve overall survival (27), although results of trials of postoperative chemotherapy vary substantially. In fact, in the U.S., postoperative studies have failed to demonstrate any benefit, whereas Japanese data favor adjuvant chemotherapy after D2 dissection (28).

Despite the significant improvements obtained from chemotherapy and chemoradiotherapy regimens, the prognosis for patients with advanced gastric cancer remains poor, with a median survival of <12 months, mainly because the disease is already advanced when the initial diagnosis is made. In recent years, substantially longer survival and significantly improved quality of life of gastric cancer patients have been obtained using targeted therapies (29, 30). In particular, molecular drugs targeting the human epidermal growth factor receptor (ErbB) family have been amply investigated and are currently under evaluation in several phase III clinical trials.

### ErbB Family

The epidermal growth factor receptor family consists of four related receptor tyrosine kinases: EGFR (ErbB1, HER1), ErbB2 (HER2, *neu* in rodents), ErbB3 (HER3), and ErbB4 (HER4) (31). Although the human ErbB genes are found on four different chromosomes, all members share a common structure, including an extracellular domain, lipophilic transmembrane region, intracellular domain containing tyrosine kinase, and a carboxy-terminal region. EGFR, the first member of this receptor family to be discovered (32), was also the first receptor for which evidence emerged of a relationship between receptor overexpression and cancer (33). Several alterations in ErbB family members were subsequently found to be correlated with the development and progression of numerous human cancers, e.g., non-small cell lung cancer (34), breast (35), colorectal (36), laryngeal (37), esophageal (38), ovarian (39), and prostate cancer (40), and melanoma (41) as a result of their pivotal role in signal transduction. In particular, the ErbB signaling network consists of several overlapping and interconnected modules including the phosphatidylinositol 3-kinase (PI3K)/Akt (PKB) pathway, the Ras/Raf/MEK/ERK1/2 pathway, and the phospholipase C (PLCγ) pathway. The PI3K/Akt pathway plays an important role in mediating cell survival, while the Ras/ERK1/2 and PLCγ pathways are involved in cell proliferation (42). These and other ErbB signaling modules influence angiogenesis, cell adhesion, cell motility, development, and organogenesis (43).

The ligands that bind to each monomeric receptor are shown in Table 1. Notably, 7 growth factors bind to EGFR, none binds to HER2, 2 bind to HER3, and 7 bind to HER4. The 4 ErbB family members form 28 homo- and heterodimers. The 11 growth factors in the EGF-like family and the 28 dimers make 614 receptor combinations possible. The binding of ligands to the extracellular domain of EGFR, HER3, and HER4 leads to the formation of kinase-active hetero-oligomers (31). The activation of HER2 and EGFR results in transphosphorylation of the ErbB dimer partner, stimulating intracellular pathways including RAS/RAF/MEK/ ERK, PI3K/AKT/TOR, Src kinases, and STAT transcription factors (42). In particular, HER2 does not bind directly to any ErbB ligand but rather is fixed in a conformation resembling a ligand-activated state, favoring dimerization (44, 45). In fact, although EGFR, HER3, and HER4 are activated by ligand binding, the specific ligands to which HER2 binds have still not been identified (46). However, aberrant HER2 activity and HER2 receptor activation results in receptor dimerization (e.g., HER2/HER3), which triggers a complex signal transduction cascade, modulating survival, proliferation, mobility and cancer cell invasiveness (47).

**Table 1:** Pattern of ErbB receptor binding.

**Growth factor**	**Receptor binding**			
	**EGFR**	**HER2**	**HER3**	**HER4**
Epidermal growth factor (EGF)	+	–	–	–
Epiregulin	+	–	–	+
Epigen	+	–	–	–
Betacellulin	+	–	–	+
Heparin-binding epidermal growth factor (HB-EGF)	+	–	–	+
Transforming growth factor-α	+	–	–	–
Amphiregulin	+	–	–	–
Neuregulin 1	–	–	+	+
Neuregulin 2	–	–	+	+
Neuregulin 3	–	–	–	+
Neuregulin 4	–	–	–	+

The HER3 receptor, despite showing weaker kinase activity than that of its ErbB co-receptors, plays a key role in promoting cell survival (48). HER3 binds ATP and catalyzes autophosphorylation. After transphosphorylation by another ErbB family member, HER3 acts as an efficient phosphotyrosine scaffold, leading to strong downstream signaling activation. In particular, HER3 is a powerful inducer of the PI3K/Akt pathway through six consensus phosphor-tyrosine sites on its C-terminal tail which bind the PI3K p85 subunit (49–51). Furthermore, in HER2-driven tumors, the HER2-HER3 dimer has proven essential for tumor formation and maintenance (52, 53). In particular, the role of HER3 in resistance to HER2-targeted therapy in this tumor subtype has been underlined in numerous studies showing that HER3 upregulation may induce resistance to several signaling inhibitors designed to directly or indirectly antagonize activated PI3K signaling. Furthermore, although the HER2-HER3 dimer is the strongest HER family dimer, HER3 has been seen to dimerize with EGFR and with non-ErbB family members, including c-MET (54, 55).

HER4 is a unique cell surface receptor that mediates the activity of transmembrane tyrosine kinase. Unlike other ErbB receptors, there is evidence that HER4 is characterized by antiproliferative and pro-apoptotic activity (56, 57). In cell line experiments when HER2-positive cancer cells were transfected to overexpress HER4, researchers observed reduced proliferation and increased apoptosis (56), suggesting that HER4 antagonizes HER2 signaling activity (58). Four HER4 receptor isoforms resulting from the alternative splicing of *HER4* mRNA have been described (JMa or JMb, Cyt1 or Cyt2) (59). The JMa isoform comprises an extracellular proteolytic site cleaved by the metalloproteinase tumor necrosis factor-alpha converting enzyme (TACE) (60). After cleavage, the transmembrane cleavage product (m80) undergoes a second intramembrane-secretase cleavage that releases a soluble HER4 intracellular domain (4ICD) into the cytoplasm (61). The 4ICD either remains in the cytosol or translocates to the nucleus. The HER4 intracellular domain is characterized by multiple biological activities and cellular responses including differentiation, pro-apoptotic pathway activation, cell cycle arrest, transcription modulation through the formation of complexes with transcription factors, and cell proliferation. These responses are associated with 4ICD localization in different cell compartments (62). Nuclear 4ICD has been found to be a powerful ER co-activator, interacting directly with ligand-associated ER and promoting ER-positive breast tumor cell proliferation (63). It has also been seen that 4ICD accumulates within the mitochondria, promoting tumor cell apoptosis through the activity of the cell-killing BH3 domain (57). The manipulation of 4ICD cell localization is thus a potentially effective therapeutic strategy.

### ErbB Expression and Gastric Cancer

EGFR is overexpressed in 27%−64% of gastric tumors (64, 65) and its role as an oncogene in this malignancy is well-known. However, there is no general consensus on the prognostic value of EGFR status in gastric cancer patients. Some authors suggest that high gene amplification is associated with poor outcome (66, 67), while others sustain the opposite (68). Moreover, a 2013 meta-analysis comparing the results obtained in 5 different studies on a total of 1,600 patients concluded that EGFR expression is not an independent predictor of survival in gastric cancer (69).

*HER2* overexpression/amplification varies considerably among studies (6 to 30%) and is partly attributable to variability in histologic subtype and primary tumor localization (70). The highest expression rates have been seen in intestinal type tumors located proximally to the gastroesophageal junction (71). Studies on gastric cancer have obtained inconsistent results on the prognostic role of HER2. Although the majority reported that positivity to HER2 is associated with a poor prognosis (72, 73), some did not observe an association between HER2 status and outcome (74) or a longer median overall survival in patients with HER2-positive gastric cancer compared with those with HER2-negative tumors (74, 75). Although the correlation between HER2 status and gastric cancer prognosis is still open to debate, HER2 protein expression or gene amplification is currently used as a biomarker for targeted therapy in this tumor (71–76).

HER3 and HER4, like EGFR and HER2, have also been found to be expressed in 20.7% and 13.3% of gastric cancers, respectively (77). A correlation between high HER3 expression and poor survival has been described in several studies (78–80). Conversely, the few studies performed to date on HER4 in gastric cancer have not clarified its role as a prognostic marker. A recent work by He et al. highlighted an association between high HER4 expression and TNM (Tumor-Nodes-Metastasis) but not HER4 overexpression and survival (77).

### ErbB Testing in Gastric Cancer

Several studies have been conducted on EGFR expression in gastric cancer, and variations in the reported expression of the receptor may be due to differences in sample size, detection methods or evaluation standards used. Some authors observed that EGFR was not expressed in normal gastric mucosa but highly expressed in gastric cancer tissue, concluding that EGFR expression could be related to *EGFR* gene amplification and mutation, continuous EGFR activation, and activation of an abnormal signal transduction pathway. However, it has yet to be proven that high *EGFR* expression in gastric cancer is a result of gene amplification or mutation.

EGFR expression can be detected in several ways, e.g., by genomic assays that quantify the number of *EGFR* gene copies or number of cell surface receptors (Table 2). Genomic assays include:

1. Fluorescence *in situ* hybridization (FISH) and chromogenic *in situ* hybridization (CISH), both of which measure *EGFR* gene amplification by quantifying gene copy number (66);
2. Immunohistochemistry (IHC), which measures the number of cell receptors, thus enabling quantification of receptor overexpression (81).

**Table 2:** Evaluation of immunostaining for EGFR and HER2.

**Classification**	**IHC Score**	**EGFR**	**HER2**
Negative	0	No staining or background type staining	No staining or <10%
Negative	1+	Discontinuous membrane staining; >10%	Faint/barely perceptible >10%
Equivocal	2+	–	Weak to moderate; complete or basolateral membrane staining; >10%
Positive	2+	Weak to moderate; >10%	IHC2+ and FISH+
Positive	3+	Moderate to strong; complete membrane staining; >10%	Moderate to strong; complete or basolateral membrane staining; >10%

*IHC, immunohistochemistry; FISH, fluorescence in situ hybridization*.

The widespread use of trastuzumab in breast cancer underlines the importance of high-quality HER2 testing and scoring to ensure the accurate identification of patients most likely to benefit from this targeted therapy. HER2 testing in gastric cancer differs from that of breast cancer because of the inherent differences in tumor biology, i.e., HER2 heterogeneity (focal staining) and incomplete membrane staining are more frequent in gastric cancer. These observations have led to the development and standardization of gastric cancer-specific HER2 testing protocols which must be adhered. HER2 status is mainly assessed by IHC (which measures the number of HER2 receptors on the cell surface, thus detecting receptor overexpression) or FISH (which detects gene amplification by measuring the number of *HER2* gene copies in tumor cell nuclei) using biopsy or surgical specimens (82). However, following the results obtained in the ToGA trial, trastuzumab was approved for use in HER2-positive gastric cancer defined as IHC 3+ or, alternatively, in FISH-positive gastric cancer in the USA and Japan. Of note, in Europe HER2-positive gastric cancer is defined as IHC 3+ or IHC 2 + plus positive FISH (83) (Table 2).

Unlike EGFR and HER2, there is no standardized method for assessing HER3 status. The most widely used methods are IHC, FISH and quantitative reverse transcription polymerase chain reaction (RT-PCR), which evaluates *HER3* on the basis of messenger RNA levels (79, 84). Finally, as HER4 testing is not routinely performed in clinical practice, there is no standard method for its assessment. However, the method most widely used in clinical trials is IHC (77).

### Agents Targeting the ErbB Family in Advanced Gastroesophageal Cancers

**Figure 1:**
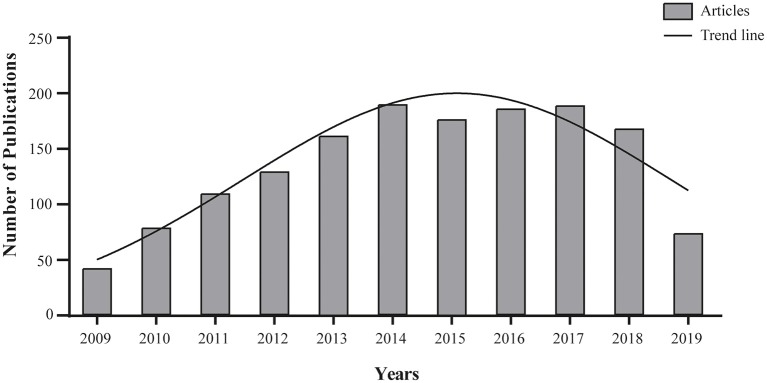
Agents targeting the ErbB family. Search for article appearing in PUBMED database over the past 10 years using the mesh terms “Stomach Neoplasms” AND “ErbB Receptors” in the Advance research builder option.

Chemotherapy is the cornerstone of treatment for locally advanced and metastatic gastroesophageal cancer. Targeted therapies, in particular those directed against ErbB family receptors, have been investigated in the preclinical setting and some are currently undergoing assessment in clinical trials (Figure 1). However, unlike EGFR and HER2, relatively little is known about the role of HER3 and HER4 in gastric carcinogenesis or about the relationship between HER3 and HER4 and clinical pathological features, including overall survival. In particular, a better understanding of HER3 receptor functionality has unveiled the molecular cornerstones of its complex mechanism of action that are targetable through multiple pharmacological strategies, i.e., inhibition of ligand binding to the extracellular domain, receptor dimerization inhibition, and inhibition of the partner tyrosine kinase activity, all of which have the potential to benefit patients with HER3 overexpressing tumors. A lasting response was obtained in a phase I trial of anti-ERBB3 mAb therapy (GSK2849330) in individuals with advanced HER3-positive solid tumors (http://www.clinicaltrials.gov/show/NCT01966445). Moreover, a phase III clinical trial (http://www.clinicaltrials.gov/show/NCT02134015) focusing on new HER3-targeted antibodies was recently concluded and results are eagerly awaited.

A phase I clinical trial (http://www.clinicaltrials.gov/show/NCT03552406) and a phase I/II clinical trial (http://www.clinicaltrials.gov/show/NCT02980341) focusing on new HER3-targeted antibodies are currently recruiting patients with different solid tumors. Furthermore, 2 phase II clinical trials (http://www.clinicaltrials.gov/show/NCT03810872 and http://www.clinicaltrials.gov/show/NCT02501603), both focusing on afatinib, are currently recruiting patients with different solid tumors. Afatinib is a promising novel small ErbB family blocker that covalently binds and irreversibly blocks signaling mediated by activated EGFR, HER2, and HER4 receptors, and also HER3 transphosphorylation. Another phase II study (http://www.clinicaltrials.gov/show/NCT01953926) is currently exploring the efficacy and safety of neratinib, an irreversible panHER inhibitor in solid tumors with activating *HER2, HER3* or *EGFR* mutations or with *EGFR* gene amplification. Confirmation of their efficacy could pave the way for their use in gastric cancer as well (Table 3).

**Table 3:** Ongoing clinical investigations of HER3 targeted agents.

**Clinical trial identifier**	**Investigational compound**	**Target**	**Phase**	**Condition**	**Status**
NCT03065387	Neratinib Palbociclib	EGFR, HER2, HER3, HER4	I	Solid tumors with EGFR mutation/amplification, HER2 mutation/amplification, HER3/4 mutation, treatment refractory and advanced or metastatic	Recruiting
CT03552406	ISU104	HER3	I	Solid Tumor	Recruiting
NCT02980341	U3-1402	HER3	I/II	Metastatic Breast Cancer	Recruiting
NCT03499626	ASLAN001	EGFR,HER2,HER3, HER4	I/II	Advanced/ Metastatic Hepatocellular Carcinoma	Recruiting
NCT01953926	Neratinib	EGFR, HER2, HER3	II	Solid tumors with somatic human epidermal growth factor receptor (EGFR, HER2, HER3) Mutations or EGFR gene amplification	Recruiting
NCT03810872	Afatinib	EGFR, HER2, HER3	II	Cancers Harboring an EGFR Mutation (Excluding Non-squamous Non- Small Cell Lung Cancer, a Registered Indication), a HER2 Mutation or a HER3 Mutation	Recruiting
NCT02501603	Afatinib	EGFR,HER2,HER3,HER4	II	Gastric Cancer, Gastroesophageal Junction Cancer	Recruiting

The following novel anti-ErbB inhibitors targeting EGFR and HER2 have been approved and are currently being developed for use in patients with gastroesophageal cancer.

### EGFR Inhibition

The most common approaches to the inhibition of EGFR require the use of monoclonal antibodies. Cetuximab (Erbitux®) is a chimeric monoclonal antibody (IgG1) that binds the extracellular domain of human EGFR, inducing its internalization, downregulation, and degradation. Furthermore, this antibody-receptor interaction competitively inhibits EGF binding, preventing receptor dimerization and blocking ligand-induced EGFR tyrosine kinase auto-phosphorylation and activation (85). Promising phase II data formed the basis for the phase III EXPAND trial (erbitux in combination with xeloda and cisplatin in advanced gastroesophageal cancer) in which 904 patients were randomized to receive cisplatin with capecitabine with or without cetuximab. However, a benefit in terms of progression-free (PFS) or overall survival (OS) was not observed in the cetuximab group (86).

RTOG 0436 is a randomized phase III trial comparing the efficacy of paclitaxel and cisplatin in combination with radiation therapy (daily 50.4 Gy/1.8 fractions) with or without cetuximab in patients with locally advanced esophageal cancer. The study is ongoing but currently not recruiting patients (https://clinicaltrials.gov/show/NCT00655876).

In contrast to their role in colorectal cancer, KRAS mutations have not proven to be a negative predictive biomarker of response to cetuximab in gastroesophageal cancer (87). Other biomarkers such as EGFR expression, copy number and phosporylation have also been evaluated, but sample size and the retrospective nature of the research have not led to meaningful conclusions (88, 89).

The other antibody used to inhibit EGFR is panitumumab (Vectibix®), the first fully human IgG2 monoclonal antibody targeting EGFR. Its activity in gastric cancer was studied in a randomized open-label multicenter trial on the efficacy of epirubicin, oxaliplatin, and capecitabine (EOX) with or without panitumumab in untreated advanced gastroesophageal cancer (REAL-3 study) (http://www.clinicaltrials.gov/show/NCT00824785). The results from this clinical trial did not show any benefit for the panitumumab-treated group, possibly due, in part, to reduced doses of chemotherapy administered in the combination arm, and the study was interrupted early (90).

In the ACOSOG Z4051 phase II study, patients with potentially resectable disease underwent neoadjuvant docetaxel, cisplatin, and panitumumab in combination with radiotherapy (91) (http://www.clinicaltrials.gov/show/NCT00757172). However, the activity of the multidrug combination was outweighed by the significant toxicity observed.

Nimotuzumab is a humanized therapeutic monoclonal antibody directed against EGFR. A phase II clinical trial is currently ongoing to assess the efficacy and safety of adding nimotuzumab to irinotecan after first-line treatment failure in patients with recurrent or metastatic EGFR-overexpressing gastric adenocarcinoma. In addition, as secondary aims, biomarkers for nimotuzumab efficacy in gastric cancer will be investigated (http://www.clinicaltrials.gov/show/NCT03400592).

Another phase II trial is ongoing to determine the safety and efficacy of varlitinib plus mFOLFOX6 for the treatment of gastric cancer (http://www.clinicaltrials.gov/show/NCT03130790). Varlitinib (also known as ASLAN001) is a small-molecule, adenosine triphosphate competitive inhibitor of EGFR, HER2 and HER4. EGFR inhibition by tyrosine kinase inhibitors (TKIs) such as iressa (Gefinitib®) and tarceva (Erlotinib®) (both oral EGFR TKIs) has also been investigated in clinical trials on gastroesophageal cancer (92, 93).

### HER2 Inhibition

On the basis of preclinical studies highlighting the significant activity of anti-HER2 therapies in both *in vitro* and *in vivo* gastric cancer models (73, 94, 95), molecular drugs targeting HER2 have been widely studied in clinical trials on gastroesophageal cancer. Trastuzumab (Herceptin^®^), a humanized monoclonal antibody that targets the extracellular binding domain of the HER2 receptor, was the first molecular targeted agent to be approved as standard treatment for gastric cancer (29, 96). It has been used in combination with cytotoxic chemotherapy in several clinical trials on gastric and gastroesophageal junction (GEJ) tumors (Table 4). The international, open-label phase III ToGA trial randomized patients with treatment-naive metastatic or locally advanced unresectable HER2-overexpressing gastric or GEJ adenocarcinoma to chemotherapy with trastuzumab or chemotherapy alone. HER2 overexpression was defined as 3+ staining by IHC or as positive FISH (29). The combination was generally well tolerated and a 2.7 month improvement in median OS was observed in the trastuzumab arm. Furthermore, response rate, time to progression and duration of response were significantly higher in the trastuzumab plus chemotherapy group.

**Table 4:** Ongoing clinical investigations of trastuzumab in gastric cancer.

**Clinical trial identifier**	**Investigational compound**	**Phase**	**Status**
NCT03680560	ACTR T Cell Product; Trastuzumab	I	Recruiting
NCT03319459	FATE-NK100; Cetuximab; Trastuzumab	I	Recruiting
NCT02805829	Trastuzumab; NK cells	I/II	Not yet recruiting
NCT02901301	Pembrolizumab; Trastuzumab; Capecitabine; Cisplatin	I/II	Recruiting
NCT01191697	Bevacizumab; Trastuzumab; Oxaliplatin; Capecitabine	II	Active, not recruiting
NCT03766607	Trastuzumab; Ramucirumab; Paclitaxel	II	Not yet recruiting
NCT02954536	Pembrolizumab; Trastuzumab; Capecitabine; Cisplatin; Oxaliplatin; 5-FU	II	Recruiting
NCT03588533	Trastuzumab; Capecitabine; Cisplatin	II	Recruiting
NCT04014075	Trastuzumab; Deruxtecan	II	Not yet recruiting
NCT02205047	Cisplatin; 5-FU; Capecitabine; Trastuzumab; Pertuzumab	II	Recruiting
NCT02678182	Capecitabine; MEDI4736; Trastuzumab; Rucaparib	II	Recruiting
NCT03556345	RC48-ADC	II	Recruiting
NCT02581462	FLOT; Herceptin; Pertuzumab	II/III	Active, not recruiting
NCT01774786	5-FU; Capecitabine; Cisplatin; Pertuzumab;Trastuzumab	III	Active, not recruiting
NCT03615326	Pembrolizumab; Cisplatin; 5-FU; Oxaliplatin; S-1; Capecitabine; Trastuzumab	III	Recruiting
NCT02578368	5-FU; Leucovorin; Oxaliplatin; Docetaxel; Trastuzumab	III	Recruiting

The HELOISE trial was a randomized, multicenter, international phase IIIb study comparing the effectiveness and safety of 2 trastuzumab dosing regimens in combination with cisplatin/capecitabine in patients with metastatic gastric or GEJ cancer (http://www.clinicaltrials.gov/show/NCT01450696). This study was interrupted for futility on the basis of results from the pre-planned interim analysis confirming the standard trastuzumab dose with chemotherapy as the standard-of-care for first-line treatment.

NCT01130337 is a sponsored phase II clinical trial designed to evaluate the disease-free survival rate of a combination of capecitabine and oxaliplatin with trastuzumab administered pre-surgery in patients with resectable gastric cancer. If a complete (R0) or microscopic residual tumor (R1) resection is obtained, patients receive a further two cycles of treatment. Trastuzumab is continued for a maximum of 1 year (available online: http://clinicaltrials.gov/show/NCT01130337, results not yet posted).

Another sponsored phase II trial (TOXAG) has recently concluded proving that trastuzumab in combination with capecitabine, oxaliplatin and radiotherapy in the adjuvant setting for gastric or gastroesophageal junction adenocarcinoma is safe and tolerable (http://www.clinicaltrials.gov/show/NCT01748773).

The Her-FLOT phase II study was designed to assess the efficacy of perioperative treatment based on trastuzumab in combination with FLOT (5FU, leucovorin, docetaxel, and oxaliplatin) in patients with HER2-positive locally advanced esophagogastric adenocarcinoma. Patients were administered trastuzumab with FLOT for four cycles prior to surgical resection followed by a further four cycles of chemotherapy with trastuzumab and nine additional cycles of trastuzumab alone. The aim of the study was to determine the rate of complete pathological response (http://www.clinicaltrials.gov/show/NCT01472029, results have yet to be posted).

RTOG 1010 is an ongoing phase III trial in which patients with locally advanced HER2-overexpressing esophageal or GEJ adenocarcinoma are randomized to receive combination treatment comprising radiotherapy, paclitaxel and carboplatin with or without trastuzumab prior to surgery (http://clinicaltrials.gov/show/NCT01196390, study is active but currently not recruiting).

What is emerging from these studies is that a growing number of patients are experiencing resistance to trastuzumab (97). This has aroused great interest in second- generation HER2-targeting agents such as pertuzumab (Perjeta^®^). Pertuzumab binds a distinct site on the HER2 receptor (extracellular domain II) and disrupts HER2 dimerization, subsequently blocking downstream signaling (98). On the basis of pre-clinical studies on GEJ and of the effectiveness of the trastuzumab and pertuzumab combination in breast cancer (99), the JACOB phase III study was designed to investigate the efficacy and safety of pertuzumab in patients with HER2-positive metastatic or locally advanced unresectable GEJ or gastric cancer receiving first-line treatment with cisplatin, fluoropyrimidine (5-fluoruracil or capecitabine) and trastuzumab (https://clinicaltrials.gov/show/NCT01774786, study is active but currently not recruiting).

Trastuzumab emtansine (TDM-1, KadCyla^®^) is an antibody-drug conjugate of trastuzumab and DM1 (derivative of maytansine, a macrolide isolated from plants), a powerful microtubule inhibitor. In preclinical gastric cancer models, TDM-1 has demonstrated more aggressive antitumor activity than trastuzumab (100). A multicenter adaptive phase II/III of TDM-1 recruited patients with HER2-positive advanced gastric cancer in progression after first-line treatment (http://www.clinicaltrials.gov/show/NCT0164tab1939). In particular, patients with higher HER2 expression experienced a better treatment effect from TDM-1than those with lower HER2 expression (101).

Another approach to targeting HER2 is through inhibition by TKIs. Lapatinib (Tykerb® (USA)/ Tyverb® (Europe) is an oral small molecule dual TKI of EGFR and HER2 that inhibits the activation of PI3K and Ras pathways, which is activation, dependent on both receptors, leading to the downregulation of receptor tyrosine kinase phosphorylation in cancer cells. Lapatinib was evaluated in combination with standard chemotherapy in patients with HER2-positive gastric and GEJ adenocarcinomas (phase III LOGIC study, http://www.clinicaltrials.gov/show/NCT00680901). This international multicenter trial investigated whether the addition of lapatinib to a capecitabine plus oxaliplatin regimen would extend the time to progression and OS. Although the trial did not meet its primary endpoint of improved OS, some subgroups (the Asian population and patients <60 years of age) were shown to benefit.

## Conclusions

Several clinical trials using ErbB receptor family targeted treatment strategies have been carried out over the past few years, with varying results. Others are currently ongoing, as extensively described in the present review. The ToGA study paved the way for the use of ErbB receptor family targeted treatments, showing that trastuzumab improves survival in HER2-overexpressing advanced gastric cancer patients. This monoclonal antibody is now acknowledged as the standard first-line treatment in this subset of patients. The role of combinations of anti-ErbB drugs and cytotoxic therapies is currently being explored in the area of advanced gastric cancer in an effort to prevent or delay drug resistance. On the other hand, drugs targeting EGFR have not repeated the encouraging results seen in early clinical trials. Similarly, lapatinib, a dual TKI of EGFR, and HER2, failed to induce a benefit in patients enrolled onto two large phase III trials. The modest efficacy of these agents may be attributable to acquired resistance or to an mismatched combination with known cytotoxic agents. Furthermore, the clinical data collected to date on molecular drugs directly targeting HER3 suggest a limited potential of these agents for the treatment of gastric cancer. However, several clinical trials are still ongoing.

It is now clear that results can only be improved by taking into account a number of important issues. First, the effects of the targeted therapy may be weakened because of differences in tumor histology (biomarkers), etiology (gastroesophageal reflux/Barret's esophagus, *H. Pylori* infection, alcohol and hot liquid intake, and smoking), and heterogeneity. Furthermore, tumor site (gastric, GEJ or esophagus) and population (Asia, America, Europe) should be considered background variables. For these reasons it is crucial to characterize tumors using established biomarkers, even though the diversity of molecular alterations acquired during malignant transformation, recurrence or metastasis makes it difficult to incorporate biomarkers into clinical trials. Finally, a better understanding of the complex interplay between growth factors and signaling pathway cross-talk would play a fundamental role in helping to identify individual patients who could benefit from ErbB receptor family targeted therapies.

## Core Tip

Despite substantial improvements in targeted therapies for advanced gastric cancer, median overall survival remains <12 months. A better understanding of the molecular pathways associated with gastric cancer carcinogenesis could lead to new and better targeted treatment options. In particular, there is increasing evidence of the important role played by ErbB family members in driving gastric cancer growth. Our paper provides an overview of published and ongoing clinical studies evaluating the antitumor potential of molecular drugs targeting EGFR and HER2.

## Author Contributions

CA and AT reviewed the literature and co-drafted the manuscript. SP performed the literature research. All authors read and approved the final version of the paper.

### Conflict of Interest

The authors declare that the research was conducted in the absence of any commercial or financial relationships that could be construed as a potential conflict of interest.
